# Evaluation of *Streptococcus uberis* Surface Proteins as Vaccine Antigens to Control *S. uberis* Mastitis in Dairy Cows

**DOI:** 10.3390/vaccines9080868

**Published:** 2021-08-05

**Authors:** Oudessa Kerro Dego, Raul Almeida, Susan Ivey, Getahun E. Agga

**Affiliations:** 1 Department of Animal Science, The University of Tennessee, Knoxville, TN 37996, USA; ralmeida@utk.edu (R.A.); ivey@utk.edu (S.I.); 2 Food Animal Environmental Systems Research Unit, Agricultural Research Service, U.S. Department of Agriculture, Bowling Green, KY 42101, USA; getahun.agga@usda.gov

**Keywords:** *Streptococcus uberis*, dairy cow, intramammary infection, surface proteins, immune response, immune reactive, western blot, protein electrophoresis

## Abstract

There is no effective vaccine against *Streptococcus uberis* mastitis in dairy cows. Objectives of this study were (1) to extract *S. uberis* surface proteins (SUSP) and determine immunoreactivity in vitro and (2) immunogenicity and efficacy in vivo. SUSP was extracted from *S. uberis*, and their immunoreactivity was tested by western blot. In total, 26 Jersey dairy cows were randomly divided into four groups. Groups 1, 2, and 3 were vaccinated subcutaneously with 4 mg, 1 mg, and 100 μg of SUSP, respectively, with Freund’s incomplete adjuvant. Group 4 (control) was injected with placebo. *S. uberis* UT888 was infused into two contralateral quarters of each cow during early lactation. Somatic cell count (SCC), bacteria count in milk, and mastitis were monitored. Our results show that SUSP contains multiple protein bands, that ranged from 10 to 100 kDa. All vaccinates showed an increased anti-SUSP IgG antibody. The SCC of all experimentally infected quarters increased after challenge but slightly decreased after day 3 with no significant difference among groups. Milk bacterial count was significantly (*p* < 0.05) reduced in high and medium doses vaccinated groups than low and control groups. In conclusion, SUSP vaccine is immunogenic and showed a promising efficacy to control bovine *S. uberis* mastitis.

## 1. Introduction

*Streptococcus uberis* is frequently isolated from dairy farm environment [1] and remarkably adaptable to environmental changes. *S. uberis* causes intramammary infection (IMI) of lactating, dry, heifers, and multiparous cows throughout the year. *Streptococcus uberis* IMI can become clinical or subclinical mastitis which, in some cases, may lead to persistent IMI without an increase in the somatic cell count (SCC) [2]. Some studies showed that *S. uberis* strains from IMI are host-adapted and have characteristics of contagious nature, whereas other strains are not host-adapted and cause transient IMI of environmental nature [1,2].

Despite several years of vaccine trials, there is no effective commercial vaccine against *S. uberis* mastitis in dairy cows that can prevent clinical disease and associated production losses. The only commercial vaccine UBAC^®^ *S. uberis* vaccine (UBAC^®^) (Laboratory of Hipra, S. A. Amir (Girona), Spain) that exists in Europe, Canada, and few other countries achieved some level of efficacy (partial efficacy) but does not prevent clinical disease and production losses [3]. The UBAC^®^ *S. uberis* vaccine from Hipra is also not well characterized under controlled experimental studies and field-based studies to confirm label claims from the producer. Intramammary vaccinations of dairy cows with bacterin induced protection from experimental infection with the same strain [4] but were less effective when challenged with different strains. Subcutaneous injection of live *S. uberis* with subsequent booster injection with *S. uberis* cell surface proteins through intramammary route induced protection against homologous strain but the efficacy was limited against heterologous strain [5]. Vaccination with multifunction protein (adhesin and glycolytic) *S. uberis* glyceraldehyde-3-phosphate dehydrogenase C (GapC) reduced inflammation post-challenge [6]. Some of the technical aspects of the challenge protocols [6] were questionable [7]. Although GapC is a highly immunogenic protein, its protective effect as vaccine antigen is yet to be determined. The *pau*A gene is a plasminogen activator [8] that was expected to promote bacterial invasion into a host tissue [9]. A vaccination trial with PauA protein induces increased antibodies that achieved partial protection [10]. The growth of *pau*A deletion mutant clone of *S. uberis* in milk or its ability to infect the udder of lactating dairy cows did not change. It was concluded that PauA protein does not have a role in the *S. uberis* IMI [11]. *S. uberis* has multiple proteins on its cell surface including *S. uberis* adhesion molecule (SUAM) and extracellular proteins that bind to host matrix, which allows bacterial adhesion and invasion of udder tissue, resulting in the establishment of IMI [12,13,14,15,16,17,18,19]. Recombinant SUAM (rSUAM) based vaccine efficacy trials in dairy cows induced good immunological responses in vaccinates compared with unvaccinated controls [20]. Under in vitro study, the hyperimmune serum from rSUAM vaccinated cows reduced *S. uberis* attachment and internalization into epithelial cells [20]. Intramammary infusion of *S. uberis* co-incubated with hyperimmune serum from rSUSAM vaccinates reduced the severity of the disease [21]. The *sua* gene mutant clone is less virulent to epithelial cells [22] and cows [16]. Series of controlled experimental vaccination of cows with rSUSAM and subsequent challenge with heterologous strains showed that rSUAM is immunogenic [20]. Similarly, intramammary administration of *S. uberis* preincubated with hyperimmune serum from rSUAM vaccinated cows reduced clinical mastitis and bacterial shedding through milk, post-challenge [21].

Vaccination trials with a single protein (subunit vaccine), such as *S. uberis* glyceraldehyde-3-phosphate dehydrogenase C (GapC) [6], plasminogen activator A (*pau*A) [10], rSUAM [20] induced increased immunity but the protective efficacy of induced immunity against *S. uberis* mastitis is limited. Recently, Collado et al. [3] developed a *S. uberis* vaccine (UBAC^®^) (Laboratory of Hipra) which reduced the clinical severity of *S. uberis* mastitis, but the induced adaptive immunity was not shown in their report.

There is no efficacious vaccine against *S. uberis* mastitis in dairy cows. Consequently, the control of *S. uberis* mastitis is based on good management practices, such as maintaining clean and dry housing areas, culling chronic cases, dry cow therapy, and treating clinical cases. The search for an effective vaccine that prevents clinical mastitis and associated production losses needs to be based on a profound understanding of humoral and cellular intramammary immunity and knowledge of virulence traits of *S. uberis*.

Virulence factors of *S. uberis* are not well understood. Encapsulation and biofilm formation [23,24,25,26,27,28], adherence and invasion of mammary epithelial cells [12,14], virulence-associated immunogenic surface proteins [29], and other virulence factors [30,31,32] appear to be associated with the disease process. An exhaustive search for a single virulence factor as a vaccine candidate seems to be of limited success. Initial host-*S. uberis* interactions induce the expression of virulence-associated immunoreactive surface proteins [29]. Based on this finding, we believe that control of *S. uberis* mastitis could be achieved by using *S. uberis* surface-associated proteins (SUSP) as a vaccine to enhance the intramammary immunity more effectively than a single protein (subunit vaccine).

The IMI by *S. uberis* usually increases in the early dry period, which often became a clinical disease during the early lactation. We hypothesize that vaccination of dairy cows during the late lactation to transition period with SUSP reduces the establishment of *S. uberis* IMI and consequently prevents clinical mastitis and associated production losses during early lactation. The objectives of this study were (1) to extract *S. uberis* surface proteins (SUSP) and test their immunoreactivity in vitro and (2) to determine its immunogenicity and efficacy against experimental *S. uberis* infection during early lactation under in vivo condition.

## 2. Materials and Methods

### 2.1. Extraction of SUSP

SUSP was extracted using a method previously developed by our group [33] with slight modifications. Briefly, nine *S. uberis* strains isolated from cases of mastitis in the United States and other countries were used. One pure colony was inoculated into 500 mL Todd Hewitt broth (THB) (BD and Company, Sparks, MD, USA) and incubated at 37 °C, and grown to mid-log phase. All subsequent procedures were the same as that of Abdi et al. [33].

### 2.2. Evaluation of SUSP by Sodium Dodecyl Sulfate-Polyacrylamide Gel Electrophoresis (SDS-PAGE) 

The SDS-PAGE protocol described by Abdi et al. [33] was used. Briefly, 20 µg of SUSP in 10 µL of phosphate buffered saline (PBS 7.4) was mixed with 10 µL of 2× Laemmli buffer with 5% beta-mercaptoethanol (Bio-Rad Lab., Hercules, CA, USA). The mixture was heated at 95 °C for 5 min, and samples were run in a 12% protein gel (Bio-Rad) [34,35] at 110 V with the constant current for 90 min. Protein bands were stained with silver stain (Bio-Rad), and images were captured using a ChemiDoc™ Touch Imaging System (Bio-Rad) and analyzed by Image Lab Software (Bio-Rad). 

### 2.3. Evaluation of SUSP by Western Blot

Western blot was conducted as described by Abdi et al. [33]. Briefly, the SUSP were separated by electrophoresis and transferred to nitrocellulose membrane (Bio-Rad) at a constant 100 V with 400 mA for 60 min by a wet method (Bio-Rad). The membrane was blocked overnight, washed, and incubated with hyperimmune serum from a cow previously vaccinated with SUSP and protected from mastitis upon challenge in another previous pilot study. The membrane was washed and incubated with horse radish peroxidase (HRP)-conjugated sheep anti-bovine IgG (H+L) secondary antibody (Bethyl Lab. Montgomery, TX, USA). The precision protein Strep Tactin-HRP conjugate (Bio-Rad) was used as a secondary antibody for molecular weight markers. Finally, the membrane was washed and 25 mL of peroxidase substrate (TMB membrane HRP substrate (SeraCare Life Sciences Inc, Milford, MA, USA) was added, and the reaction was fully developed at room temperature. Protein band images were taken using a ChemiDoc™ Touch Imaging System (Bio-Rad) and analyzed using Image Lab Software (Bio-Rad). Images on the gel and the membrane were compared to identify immune-reactive protein bands.

### 2.4. Identification of Immunoreactive Proteins in the SUSP from S. uberis UT888 by Sequencing from Gel Band 

After visually identify bands that contain proteins, each visible band was excised using a clean scalpel blade and placed in a sterile Eppendorf tube with laboratory-grade sterile water, and submitted to Bioproximity, http://www.bioproximity.com, accessed on 4 January 2021, (Bioproximity, LLC, Chantilly, VA, USA), for protein analysis by liquid chromatography-tandem mass spectrometry (GeLC-MS/MS).

### 2.5. Evaluation of Immune Responses Induced by SUSP Vaccine

#### 2.5.1. Experimental Animals and Vaccination Protocol

A total of 26 Jersey breed dairy cows from Middle Tennessee AgResearch and Education Center (MTREC; Lewisburg, TN, USA), in their 1st or 2nd lactations that were free of IMI and had low titer against *S. uberis* UT888 were divided randomly into four groups. Experimental cows were kept under similar management with other cows in the farm and usually fed a mixed total ration and other supplements required for dairy cows following the normal standard operating protocol of the farm. The experimental animals were milked last. Cows in Groups 1 (*n* = 6, High dose), 2 (*n* = 6, Medium dose), 3 (*n* = 7, Low dose) were vaccinated subcutaneously with 4 mg, 1 mg, and 100 µg of SUSP from *S. uberis* UT888 in the total volume of 5 mL containing Freund’s incomplete adjuvant (FIA). Cows in Group 4 (*n* = 7, control) were injected with PBS (pH 7.4) with FIA (Table 1). Cows were selected based on the bacteriological and clinical data obtained during the previous 6 months to the initiation of the study. Cows are also screened for serum titer against *S. uberis* UT888 by enzyme-linked immunosorbent assay (ELISA) at three weeks before vaccination. Cows that had severely damaged teat ends or had extended dry periods (>90 days) were not included in the study. Three series of vaccinations were given at drying off (D0), 28 days after drying off (D28), and at 7 days after calving (C7). The first vaccination was given on the upper left side of the neck, the second on the right side, and the third was on the lower left side of the neck. Vaccines were masked to personnel administering the vaccinations and subsequently conducting the assays. Treatment codes were uncoded after the completion of data collection and sample analyses. Injections were given using sterile disposable syringes and needles. This study was approved by The University of Tennessee, Institutional Animal Care and Use Committee (IACUC) (Registration Number TN-159-DES-29, 29 December 2017).

#### 2.5.2. Clinical Assessment of Animals Following Vaccination 

Animals were examined, and rectal temperatures were taken 24 h before and immediately before vaccination and then at 24 h intervals daily for 4 days, and at 7- and 14-days post-vaccination. Vaccine injection site reactions were measured at the same times described above. If swelling was noted, the length (cranial/caudal), width (dorsal/ventral), and height (thickness) were measured in centimeters (cm) using the Caliper (Thermo Fisher Scientific, Waltham, MA, USA). Animals were examined for evidence of systemic reactions such as lameness, anorexia, rapid respiration, and anaphylaxis before vaccination, within an hour after, and approximately 24 h following each vaccination.

#### 2.5.3. Sample Collection

Blood samples (10 mL) for evaluation of immune responses were collected immediately before each vaccination at drying off (D0), 14 days after drying off (D14), 28 days after drying off (D28), 42 days after drying off (D42), calving (C), 7 days after calving (C7), 14 days after calving (C14), 28 days after calving (C28) or immediately before challenge (Ch0), 7 days after challenge (Ch7), 14 days after challenge (Ch14), 21 days after challenge (Ch21), and 28 days after challenge (Ch28). Composite milk samples from all quarters (50 mL) were obtained immediately before vaccination at drying-off, at calving, and at 14 and 28 days after calving. Blood and milk samples were processed into serum and skim milk, respectively, and tested for anti-SUSP IgG antibodies.

#### 2.5.4. Evaluation of Anti-SUSP IgG Antibody in Milk and Blood

Antibodies in blood and milk were measured by ELISA as described [36] and only serum titers were presented since the complete analysis of anti-SUSP IgG antibody response for all time points was completed only for serum samples. Briefly, antibody response to the SUSP was analyzed by indirect ELISA. The secondary antibody was sheep anti-bovine IgG conjugated with HRP (Bethyl Lab). The absorbance was read at 405 nm with a Synergy H1 plate reader (Biotek instrument Inc., Winooski, VT, USA). Data were exported to Excel (Microsoft Corporation, Redmond, WA, USA).

### 2.6. Experimental Infection and Assessment of Efficacy

#### 2.6.1. Challenge Inoculum Preparation and Challenge

The challenge strain was *S. uberis* UT888. The organism was stored at −80 °C, thawed, and inoculated to blood agar plates (BD and Company), and grown overnight (24 h) at 37 °C with 5% CO_2_ and a 95% air incubator. One day before the challenge infection, a pure colony was inoculated to 5 mL of Todd Hewitt broth (THB) (BD and Company) and grown overnight (24 h). On the challenge day, the overnight culture was diluted at 1:100 in fresh THB and grown to the OD_600_ 0.5 (mid-log phase) for 4–5 h. The culture was diluted to the approximate bacterial count of 10^3^–10^4^ CFU/mL in PBS (pH 7.2) based on previously optimized growth and dilution using viable plate count. The number of *S. uberis* in the challenge suspension was determined by viable plate count before and after intramammary administration. Cows were challenged by intramammary administration of 5 mL of 3.5 × 10^3^ CFU of *S. uberis* UT888 into two contralateral quarters of each cow within 15 min after milking at 28 days after calving. Before intramammary administration, teat openings were cleaned thoroughly with 70% alcohol, and the bacterial suspension was administered by sterile syringes attached to sterile disposable teat cannulas. The administered inoculum was pushed up the teat canal into the teat and gland cisterns. The teat end was dipped into an antiseptic solution.

#### 2.6.2. Clinical Examination of Cows after Challenge

After the challenge, the general overall health of the animal, the clinical condition of the udder, rectal temperature, milk somatic cell count (SCC), the identity and number of bacteria (in CFU/mL) shed through milk were monitored. Body temperatures were recorded 24 h before and immediately before the infection, at 24 h intervals for seven days, and at 14-, 21-, and 28- days post-challenge. The clinical status of each quarter and the appearance of milk were recorded as described by Merrill et al. [36]. Briefly, changes in milk samples were recorded as 1: normal milk, 2: alterations in milk (flakes), 3: abnormal milk (clots, clumps, changes in milk color). Changes in mammary gland tissue were scored as 1: Slight swelling; less pliable udder, 2: Moderate swelling; firm udder, the udder became red and hot, the animal had discomfort, 3: Severely swollen; the udder is very hard, red, and hot, the distinct difference compared with other udder quarters and the cow shows symptoms of irritation. Clinical mastitis was defined as udder quarters with a clinical score of 2 in milk and gland tissue or a score of 3 either in milk or in the gland tissue with the confirmation of the isolation and identification of challenge strain of *S. uberis* UT888 for three consecutive days within 7 d of challenge.

#### 2.6.3. Sample Collection

Samples of milk were obtained from all cows immediately before the challenge, daily for 7 d, and weekly for the next three weeks (14, 21, and 28 days) after the challenge. Milk samples were collected after teat openings were thoroughly cleaned and dried with an individual paper towel and wiped with 70% ethanol. Individual udder quarter milk samples (3 mL) for microbiological analysis, individual quarter milk samples (10 mL) for SCC were collected. Blood samples (10 mL) for serum preparation were collected immediately before the challenge and at 7, 14, 21, and 28 d after the challenge.

#### 2.6.4. Somatic Cell Count (SCC)

The SCC was determined at the Dairy Herd Improvement Association (DHIA) Laboratory (Knoxville, TN, USA) using the Soma Count 300 (Bentley Instruments Inc., Chaska, MN, USA). Milk yields were recorded daily for four weeks after the challenge. Average daily milk production from the week before the challenge was used as the baseline estimate of production.

#### 2.6.5. Milk Bacterial Count 

The *S. uberis* counts in colony-forming units (CFU)/mL) in milk was determined in quarter foremilk samples during the post-challenge period. The bacterial CFU/mL were determined using plate dilution techniques, and results were presented as log base 10 CFU/mL of sample. Samples of milk were evaluated following NMC’s microbiological procedures as described [37].

#### 2.6.6. Streptococcus Uberis Identification

Identification of *S. uberis* in mammary secretion samples from experimentally challenged cows was conducted as follows: isolates that were tentatively diagnosed as streptococci were further tested for growth in 6.5% NaCl, hydrolysis of esculin and sodium Hippurate, and the reaction of Christie–Atkins–Munch–Peterson (CAMP) test to detect beta-hemolytic streptococci. Streptococci were identified to the species level by API 20 Strep method (bioMerieux Vitek, Inc., Hazelwood, MO, USA). In addition, selected *S. uberis* isolates (the first and last time isolated) were evaluated by PCR-based DNA fingerprinting as described [38] (data not shown). *S. uberis* infected quarters were identified by isolation of the challenge strain from foremilk samples collected following challenge exposure. Udder quarter was considered infected if *S. uberis* was isolated on two occasions within 7 d of challenge or when clinical mastitis resulting from *S. uberis* infection was detected.

#### 2.6.7. Treatment of Cows That Developed Clinical Mastitis

Animals in which infection became clinical were treated by intramammary administration of 200 mg of cephapirin sodium or ToDAY^®^ for lactating cows (Boehringer Ingelheim Animal Health USA Inc., Duluth, GA, USA). Cephapirin sodium was chosen based on in vitro sensitivity of *S. uberis* using the broth microdilution method on commercially prepared 96-well microtiter plates for the Sensititre system (Thermo Fisher Scientific, Cleveland, OH, USA) using Clinical Laboratory Standards Institute guidelines [39]. During antibiotic treatment, milk was discarded for at least 96 h after the last treatment per the manufacturer’s recommendations. All treated cows cleared the infection.

### 2.7. Statistical Analysis

All statistical analyses were performed in STATA 16.1 (StataCorp LLC, College Station, TX, USA). Interaction terms were assessed using the likelihood ratio test starting with higher-level terms. The effect of vaccination on IgG response was assessed with a mixed-effects linear regression model. The main effects of treatment group, time, and treatment by time interaction were included as fixed effects. A cow was included as a random effect to adjust for repeated measurements within a cow over 13-time points. Mean IgG response was plotted over the time points and by the treatment groups. Bonferroni adjusted contrast values were obtained to compare the IgG values among the treatment groups at a particular time point and over time. Treatment by time interaction was significant for IgG values. Log_10_ SCC was analyzed by mixed-effects linear regression model that included the main effects of treatment, quarter level challenge status (yes or no), sampling time during the challenge, and the random effect of a cow. Three-way interaction was not significant (*p* = 0.5179); treatment by time interaction was not significant (*p* = 0.8826). The final model consisted of the three main effects and time-by-challenge interaction. The efficacy of the trial vaccine was evaluated based on the recovery of the same bacterial strain used for the challenge, counts of bacteria and somatic cell count per mL of milk, and occurrence of clinical mastitis episodes. Treatment differences in the incidence of clinical mastitis per infected quarter due to *S. uberis* are the parameter of primary interest. Intervals between vaccinations were examined for use as a possible covariant in any mixed model analyses. Clinical mastitis data were summarized using frequency tables. Bacteria counts were analyzed with Poisson regression and expressed as log_10_ CFU/mL; pairwise comparisons were made among the treatment groups. Somatic cells were log-transformed, and the data were analyzed using a mixed linear model with repeated measurements. Continuous measures were evaluated using a mixed model ANOVA testing the fixed effects of vaccination (treatment), time, and the interaction of treatment and time, and the random effect of cows grouped within a treatment. To better discern potential differences that exist in our response variables for experimental infection status (clinical, subclinical, uninfected), additional analyses were conducted that include the fixed effect of status. The *p* ≤ 0.05 was considered significant.

## 3. Results

### 3.1. Extraction of SUSP and Evaluation of Their Immunoreactivity under In Vitro Condition

**Figure 1:**
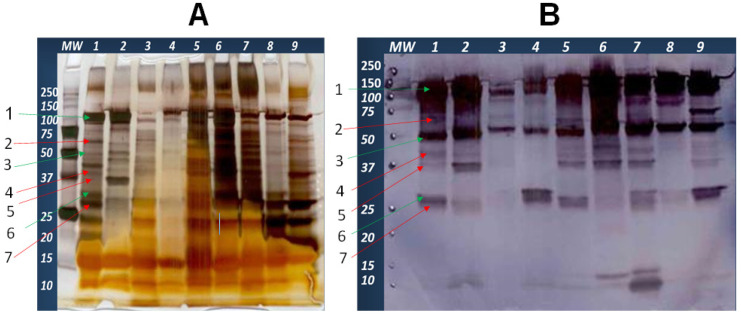
SDS-PAGE and Western blot results of SUSP extracted from nine isolates using cholic acid. Panel (**A**): Silver stained SDS-PAGE of nine different *S. uberis* isolates, MW: protein molecular weight marker, 1: UT888, 2: UT366, 3: UT754, 4: 0140J, 5: UT999, 6: UT102, 7: NZ1636 (strain from New Zealand), 8: C5207 (strain from Colorado), 9: W11020 (strain from Washington). Panel (**B**): Western blot results of the same SUSP transferred to the membrane from the gel, 1: UT888 2: UT366 3: UT754 (natural SUAM defective mutant) 4: 0140J, 5: UT999 6: UT102 7: NZ1636 8: C5207, 9: W11020.

Evaluation of SUSP after electrophoretic separation on one-dimensional gel electrophoresis (1D SDS-PAGE) showed more than 15 protein bands ranging in size from 10–100 kDa. Similarly, the Western blot result showed different protein bands of 10–100 kDa size (Figure 1A). Representative *S. uberis* strain UT888 (Figure 1A, lane 1) had about 15 visible protein bands, whereas *S. uberis* UT366 (Figure 1A, lane 2) had 13. Western blot results (Figure 1B) showed that hyperimmune serum from previously vaccinated cows reacted with SUSP from nine isolates (Figure 1B). Bands at a molecular weight of 30, 50, and 100 kDa, strongly reacted with immune serum and conserved across all 9 strains tested (Figure 1B).

Most bands were present in 9 strains of *S. uberis* evaluated. In general, SUSP contains a variety of proteins that are likely to have roles in virulence and metabolism. Some of the proteins identified by sequencing of bands from *S. uberis* UT888 (Figure 1, lane 1) were *S. uberis* adhesion molecule/protein (band #1: SUAM), lactoferrin binding protein (#2: Lbp), Glutamine synthetase (#3: GlnA), elongation factor Tu (#4: Tuf), *S. uberis* glyceraldehyde-3-phosphate dehydrogenase (#5: GapC), *S. uberis* plasminogen activator protein (#6: PauA), fructose-biphosphate aldolase (#7: FbA) (Table 2).

### 3.2. Evaluation of the Immunogenicity of SUSP in Dairy Cows 

#### 3.2.1. Safety of SUSP Vaccine

There was no adverse reaction to the vaccine. In most of the vaccinated cows, the local vaccine injection site reaction was similar to that of the control group. However, one animal from the high dose vaccinated group showed a significant site reaction than the control group, but it returned to normal values in about seven days (data not shown).

#### 3.2.2. Immunogenicity of SUSP Vaccine and Its Efficacy

**Figure 2:**
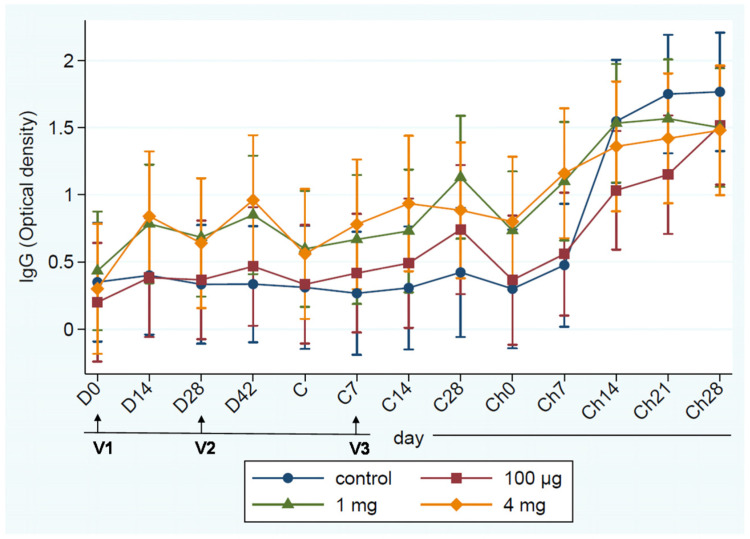
Serum anti-SUSU IgG antibody of cows vaccinated with different doses. Data are presented as a mean of each group, and error bars are 95% confidence intervals of the mean values. V1: first vaccination, V2: second vaccination, V3: third vaccination, D0: at drying off, D14: 14 days after drying off, D28: 28 days after drying off, D42: 42 days after drying off, C: at calving, C7: 7 days after calving, C14: 14 days after calving, C28: 28 days after calving, Ch0: Immediately before the challenge, Ch7: 7 days after challenge, Ch14: 14 days after challenge, Ch21: 21 days after challenge, Ch28: 28 days after challenge.

**Figure 3:**
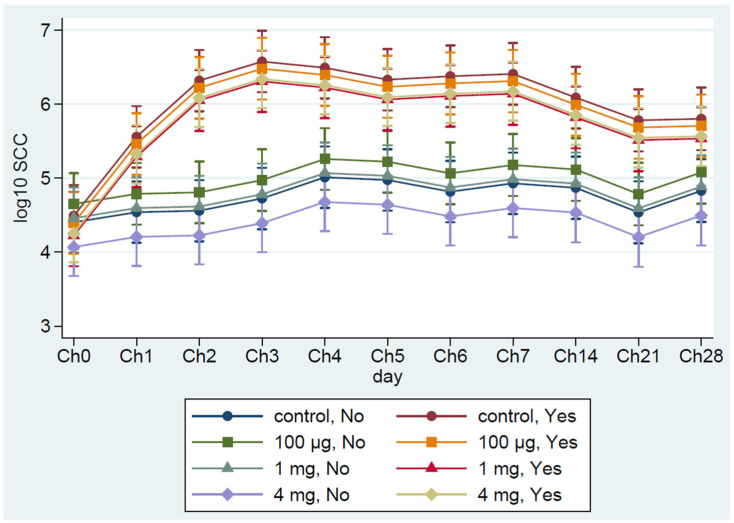
Milk somatic cell count from challenged and non-challenged quarters. Ch0: immediately before the challenge, Ch1–Ch28: Days 1–28 post-challenge, Data are presented as log_10_ of the actual counts, and error bars represent 95% confidence intervals for the mean values of all the challenged quarters (Yes) and non-challenged (No) quarters per group.

Although Bonferroni adjusted pairwise comparisons on each sampling day were not statistically significant (*p* > 0.05), cows vaccinated with high (4 mg) and medium-dose (1 mg) mounted higher immune responses than the low dose (100 µg) vaccine and the unvaccinated control (Figure 2). Serum IgG level was increased and remain sustained in the two vaccine groups (High and medium) through the two booster doses during the dry period and early lactation. However, regardless of the vaccine group, IgG response significantly (*p* < 0.001) increased following the challenge with *S. uberis* compared to the pre-vaccination level at D0. Antibody (IgG) test was conducted on both milk and serum, but complete analysis of titers at all time points was performed only for serum. Subsequently, results were shown only for serum titers. The log_10_ SCC significantly (*p* < 0.001) increased in the challenged quarters from their pre-challenge levels regardless of the treatment group. SCC in the challenged quarters was significantly (*p* < 0.05) higher than the non-challenged quarters for most of the challenging periods depending on the vaccine dose (Figure 3).

#### 3.2.3. Milk Bacterial Counts

**Figure 4:**
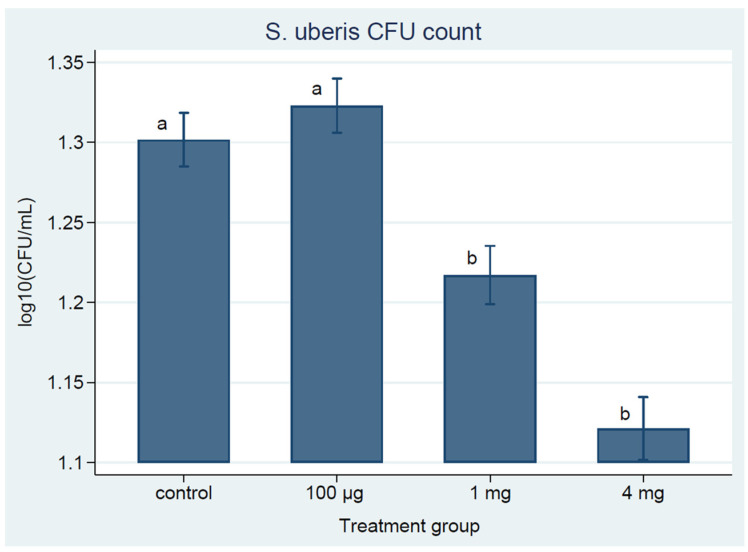
*S. uberis* UT888 counts in CFU/mL of milk from the high, medium, and low dose vaccinated and control cows. Data are presented as the mean of base 10 log CFU/mL per group at each time point and error bars represent the 95% confidence intervals. Different letters show statistically (*p* < 0.05) different counts.

**Figure 5:**
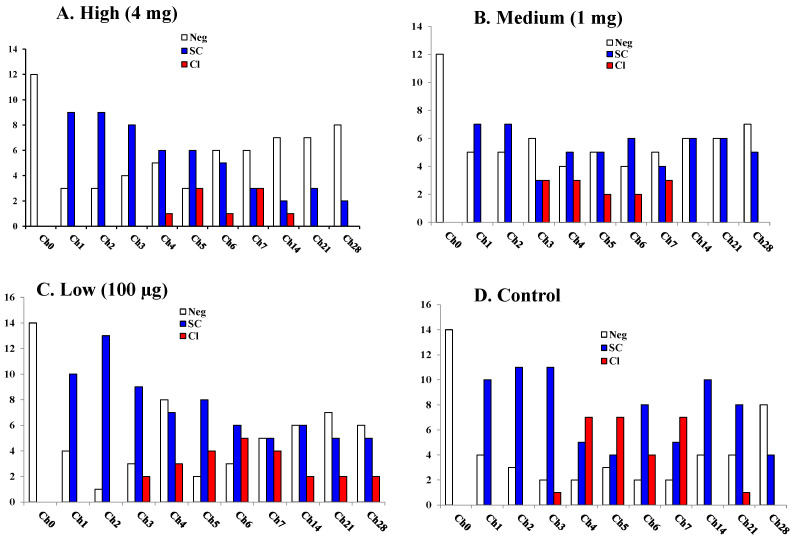
Mastitis status (clinical: Cl or Subclinical: SC or Negative: Neg) in cows vaccinated with SUSP upon experimental challenge. Panels (**A**) (high dose, 4 mg), (**B**) (medium dose, 1 mg), and (**C**) (low dose, 100 μg) showed quarter-level mastitis status of different SUSP dose vaccinated groups. Panel (**D**) (control) was the control group. Negative quarters (Neg, white bars) were those which did not show clinical mastitis scores, without isolation of challenge strain of *S. uberis* UT888 from milk. Quarters with subclinical (SC, blue bars) mastitis were those with isolation of challenge strain of *S. uberis* UT888 from milk but no visible inflammatory changes in milk and udder tissue. Quarters with clinical (Cl, red bars) mastitis were those that manifested clinical symptoms of mastitis. Data were presented as total numbers of negative, subclinical, or clinical quarters per group at each sampling time point. Ch0: Immediately before the challenge, Ch1–Ch28: days 1–28 post-challenge.

Bacteriological analysis of milk from experimentally infected quarters showed the presence of significantly (*p* < 0.05) reduced numbers of challenge strain of *S. uberis* UT888 in cows vaccinated with 1 mg or 4 mg doses than the control or the low dose groups (Figure 4). Other than challenge strain, the most frequently isolated bacteria were non-aureus *Staphylococcus* species, and their counts were variable among quarters but mostly in the range of 1 to 10 CFU/mL. Other bacteria infrequently observed were *Bacillus* species. Challenge strain *S. uberis* counts did not differ between control and low dose vaccine; similarly, it did not significantly (*p* > 0.05) differ between the medium and high dose vaccinated groups as shown in Figure 4. All groups had IMI, some of which resulted in clinical mastitis (Figure 5A–D). The more noticeable difference was detected between the high dose vaccinated and control groups. In the high dose vaccinated group, nine quarters were sub-clinically infected (SC; positive for *S. uberis* with SCC > 500,000) at Ch1. However, at Ch3, only 3 quarters were SC, and at the end of the trial (Ch28), all udder quarters were free of IMI (Figure 5A). In contrast, quarters with SC in the control group (Figure 5B) were between 7 and 8 up to Ch6, and at Ch28, only two quarters had *S. uberis* IMI. The highest clinical mastitis was observed in the control group, while the lowest was in the high dose vaccinated group.

## 4. Discussion

The SUSP was extracted from different *S. uberis* isolates from different areas using cholic acid as previously described [33] with minor modifications. The SUSP comprises different bands ranging in sizes from 10 to 100 kDa, which had a variable intensity of reactions to hyperimmune serum from a cow vaccinated with SUSP. Some of the proteins were detected by sequencing of SUSP from *S. uberis* UT888 include *S. uberis* adhesion protein/molecule (SUAM), lactoferrin binding protein (Lbp), glutamine synthetase (GlnA), elongation factor Tu (Tuf), glyceraldehyde-3-phosphate dehydrogenase (GapC), plasminogen activator A (PauA), and fructose-biphosphate aldolase (FbA), and others. The dominant proteins in the SUSP are multifunctional proteins that are mainly involved in energy metabolism and other cellular functions, as well as virulence-associated functions. Some of them are generally believed to be cytosolic proteins (e.g., GapC, Elongation factor Tu, etc.) but recently some studies showed that they are multifunctional proteins [40] which were found to be associated with bacterial cell surface as virulence factors (e.g., GapC serve as an adhesin that specifically binds to host cell surface ligand). 

SUAM plays a major role in the *S. uberis* attachment to and internalization into mammary epithelial cells. *S. uberis* ligand binds to host factors and exploits this molecular complex as a bridge to facilitate bacterial adherence to mammary cells. The rSUAM induced strong immune responses in dairy cows [20] but its potential as a protective antigen is still under evaluation.

The role of glyceraldehyde-3-phosphate dehydrogenase (GAPDH) in adhesion has been extensively documented [41,42,43,44,45,46], which showed that GAPDH is a glycolytic multifunctional protein that serves as an adhesin for colonization of tissue surfaces both for pathogenic and non-pathogenic normal microflora. GAPDH induces immune responses against *Streptococcus dysgalactiae*, *S. uberis,* and *Staphylococcus aureus* during mastitis in dairy cows. In other microorganisms GAPDH was reported to induce immune responses against *Schistosoma mansoni* in humans, *Brucella abortus* in cattle, sheep, and mice, *Candida albicans* in rabbit and *Echinococcus multilocularis* in mice [47,48,49,50,51,52,53].

Three series of vaccinations of dairy cows with SUSP at drying off (D0), 28 days after drying off (D28), and 7 days after calving (C7) with FIA induced specific anti-SUSP IgG antibody response. Freund’s adjuvant was selected with the intent to induce broader balanced and enhanced humoral and cellular immunity. Out of three different doses tested, the highest dose (4 mg) induced the highest serum anti-SUSP IgG antibody response. Subsequent experimental challenge of SUSP vaccinated cows by intramammary infusion of *S. uberis* UT888 during early lactation resulted in IMI of all cows; however, cows vaccinated with high and medium doses had the lowest *S. uberis* count in milk, number of quarters with *S. uberis* IMI, number of quarters with clinical mastitis, mastitis duration, and scores of abnormal pathological changes in milk and udder tissue compared with the control group. Similar to our findings, Collado et al. [3] reported that vaccination of dairy cows with the UBAC^®^ (Laboratory of Hipra) reduced clinical severity of mastitis but the authors [3] did not present data on the type of adaptive immunity induced by the UBAC^®^ vaccine. Similarly, Finch et al. [4] showed that intramammary vaccinations of dairy cows with *S. uberis* bacterin induced some protection from challenge with the same strain. Finch et al. [5] also showed that subcutaneous injection of dairy cows with live *S. uberis* followed by booster injection with cell surface extract from *S. uberis* prevented from challenge with the same strain but less protective against a different non-vaccine strain. It is important to mention that there were background titers in all animals but the magnitude varies with animals. We believe that this baseline titer is from previous exposure to *S. uberis* or other bacteria that are closely related to *S. uberis* or have cross-reacting epitopes. Based on our experience the background titer can be reduced to a very low level if large number of animals are screened but cannot be avoided unless pathogen free animals are used. We also did not evaluate the types of immunity (cellular or humoral) induced and detailed cytokine expression patterns induced by SUSP vaccine and further study is required to delineate the types of immunity induced by SUSP and associated cytokine expression patterns.

Somatic cell count is considered a reliable parameter for monitoring IMI, and an increase in SCC is positively associated with the severity of the infection [54,55,56,57,58]. Similarly, a decrease in SCC is considered as an indication of recovery and clearance of the infection. In this study, all the infused quarters showed an expected sudden increase in SCC in all groups, with a similar increase in antibody titer for a short period followed by a slightly decreasing trend in SCC after Ch3 but no significant difference among Groups. In agreement with the SCC, the number of quarters that showed clinical or subclinical mastitis was the lowest in the high dose vaccinated group than the medium or control group. Similarly, the greatest significant (*p* < 0.05) reduction in bacterial counts was detected in the high dose vaccinated group than the medium or control group.

These observations showed that vaccination with the medium and high doses of SUSP conferred partial protection characterized by low bacterial count shed in milk, reduced number of quarters with mastitis compared with the control group. The observed reduction in bacterial count and quarters with mastitis may be achieved by adaptive humoral (antibodies) or cellular or both arms of immunity induced by the SUSP vaccine. It is also important to mention that this partial protection was achieved against homologous strain and future study on this vaccine requires dose optimization and challenge with heterologous strain to improve the efficacy of this vaccine. A further detailed investigation is required to determine the types of adaptive intramammary immunity induced and mechanisms of its regulation including cytokine expressions associated with these observed effects.

Based on the results of experimental vaccine efficacy trials [20,52,53], bacterin vaccines induced partial protection against *S. uberis* mastitis. Contrary to bacterin vaccines, vaccination trials with a single protein (subunit vaccine) such as *S. uberis* GapC [6], pauA [10,11], SUAM [20] did not induce full protection against mastitis.

This vaccination schedule was designed to induce protection during the transition periods when dairy cows are highly susceptible to new *S. uberis* IMI. Vaccine trials with crude bacterin that contain several components of bacteria had more efficacy on homologous strain than a single purified protein. Similarly, Collado et al. [3] reported some protective effects of *S. uberis* vaccine (UBAC^®^) (Laboratory of Hipra), which contains thermostable culture supernatant of biofilm-forming strain with lipoteichoic acid as major components. It is evident, thus far, that bacterin vaccines, at best, reduce the severity of clinical mastitis. It is also apparent that a single surface protein vaccine was not effective. Our SUSP vaccine is not a single protein but a mixture of immunogenic surface proteins. Therefore, combining conserved immunogenic surface proteins at appropriate doses of each component protein with suitable adjuvants, route of injection, and vaccination time may induce effective immunity that prevents clinical disease and production losses.

## 5. Conclusions

Vaccination with SUSP increased anti-SUSP serum IgG, and subsequent experimental challenge resulted in IMI. Cows vaccinated with high and medium doses had the lowest *S. uberis* count in the milk, quarters with clinical mastitis, and mastitis duration compared with the control group. However, observed differences in the antibody responses and SCC were not statistically significant. Further vaccination regiment optimization and challenge with heterologous strain is required to improve the efficacy of this vaccine. Optimizing SUSP dose with more immunogenic proteins and appropriate adjuvant, route, and schedule of vaccination, may improve the efficacy of the SUSP vaccine.

## Figures and Tables

**Table 1:** Vaccination protocol.

Gr	# of Animals	Vaccine (SUSP) Dose	Dose/Volume	Route/Frequency
1	6	High: 4 mg in 2.5 mL of PBS	4 mg/5 mL	SC/3 times at D0, D28, C7 for all groups
2	6	Mediu: 1 mg in 2.5 mL of PBS	1 mg/5 mL	
3	7	Low: 100 μg in 2.5 mL	0.1 mg/5 mL	
4	7	2.5 mL PBS	5 mL	

Gr: Group, SUSP: *Streptococcus uberis* surface proteins, FIA: Freund’s incomplete adjuvant (2.5 mL), SC: Subcutaneous, D0: at drying off, D28: 28 days after drying off, C: calving, C7: 7 days after calving.

**Table 2:** Major immunoreactive proteins in the SUSP of *Streptococcus uberis* UT888 identified by sequencing.

Band #	Uniprot Protein ID	Mass	Log_10_E	Intensity	Peptide	Identification	Spectra	Coverage	Parsimony
1: SUAM	Q2VEB5	97.79	−32,153	2.23	195	2880	2880	90.3	differentiable
2: Lbp	Q6U7J0	62.864	−22.6	0.48	3	4	4	12.3	differentiable
3: GlnA	B9DVR7	50.49	−39.9	0.48	3	3	3	27.1	equivalent
4: EF-Tu	B9DRL9	43.91	−353	1.49	23	53	53	87.7	differentiable
5: GapC	Q8kVU6	35.9	−341.8	1.45	22	42	42	84.8	differentiable
6: PauA	Q7B2W7	33.42	−1314	1.61	36	100	100	82.5	set
7: FbA	B9DTK0	31	−296.8	1.41	22	41	41	76	differentiable

E: Expectation value in Log_10_, a value of -3 indicates a 1 in 1000 chance of occurring at random. possibility of occurring by random chance, Peptide-: spectra (number of tandem mass spectra), parsimony (protein identification by peptides), distinct—A protein is identified only by unique peptides, equivalent- a protein identified by the same set of shared peptide(s), differentiable—A protein identified by at least one shared peptide but can be distinguishable from other proteins at least by one distinct peptide, set: protein identified by a set of peptides, #1: *S. uberis* adhesion protein/molecule (SUAM), #2: Lactoferrin binding protein (Lbp), #3: Glutamine synthetase (GlnA), #4: elongation factor Tu (EF-Tu), #5: glyceraldehyde-3-phosphate dehydrogenase (GapC), #6: plasminogen activator A (PauA), #7: fructose-biphosphate aldolase (FbA).

## Data Availability

Not applicable.
